# Reliability and validity of a Mental Health System Responsiveness Questionnaire in Iran

**DOI:** 10.3402/gha.v7.24748

**Published:** 2014-07-28

**Authors:** Ameneh S. Forouzan, Hassan Rafiey, Mojgan Padyab, Mehdi Ghazinour, Masoumeh Dejman, Miguel S. Sebastian

**Affiliations:** 1 Social Determinants of Health Research Centre, University of Social Welfare and Rehabilitation Sciences, Tehran, Iran; 2 Department of Public Health and Clinical Medicine, Umeå International School of Public Health, Umeå University, Umeå, Sweden; 3 Social Welfare Management Research Center, University of Social Welfare and Rehabilitation Sciences, Tehran, Iran; 4 Ageing and Living Conditions Programme, Centre for Population Studies, Umeå University, Umeå, Sweden; 5 Department of Social Work, Umeå University, Umeå, Sweden

**Keywords:** Mental Health System, Responsiveness, Reliability, Validity, Questionnaire, Iran

## Abstract

**Background:**

The Health System Responsiveness Questionnaire is an instrument designed by the World Health Organization (WHO) in 2000 to assess the experience of patients when interacting with the health care system. This investigation aimed to adapt a Mental Health System Responsiveness Questionnaire (MHSRQ) based on the WHO concept and evaluate its validity and reliability to the mental health care system in Iran.

**Design:**

In accordance with the WHO health system responsiveness questionnaire and the findings of a qualitative study, a Farsi version of the MHSRQ was tailored to suit the mental health system in Iran. This version was tested in a cross-sectional study at nine public mental health clinics in Tehran. A sample of 500 mental health services patients was recruited and subsequently completed the questionnaire. Item missing rate was used to check the feasibility while the reliability of the scale was determined by assessing the Cronbach's alpha and item total correlations. The factor structure of the questionnaire was investigated by performing confirmatory factor analysis (CFA).

**Results:**

The results showed a satisfactory feasibility since the item missing value was lower than 5.2%. With the exception of access domain, reliability of different domains of the questionnaire was within a desirable range. The factor loading showed an acceptable unidimentionality of the scale despite the fact that three items related to access did not perform well. The CFA also indicated good fit indices for the model (CFI=0.99, GFI=0.97, IFI=0.99, AGFI=0.97).

**Conclusions:**

In general, the findings suggest that the Farsi version of the MHSRQ is a feasible, reliable, and valid measure of the mental health system responsiveness in Iran. Changes to the questions related to the access domain should be considered in order to improve the psychometric properties of the measure.

Using rigorous methods to investigate patients’ experiences and opinions when interacting with the mental health care system are recognized as important indicators of the system's performance (1). The results of such investigations can provide useful guidance for policy makers in improving mental health services (2). For instance, it is well known that there is a relationship between the overall satisfying experience with the health care system and adherence to mental health treatment (3–5). Having a better understanding of patients’ perceptions may lead to better performance and increased quality of care, as well as increased service utilization (6). Improving the quality of different aspects of the interaction between individuals and the health system can also contribute to improving the general well-being and health status of the patients (7–9).

The new era of mental health services in Iran started in 1986 after the National Program of Mental Health was adopted and implemented in the whole country (10). After a decade of program expansion, an improvement in the mental health service was achieved (11). The coverage of mental health services improved and both active screening and active follow-up of the patients, especially in rural areas, were developed (12). Although the service coverage in urban areas was still around one-third of the population, by 2006, evaluations showed that mental health program coverage reached 82.8% of the rural population (10). Despite this considerable effort, there is still need for improvement in terms of evaluation and monitoring of the quality of services provided (11). One of the areas in need of expansion is the focus on service users’ experiences, as this can offer reliable and valid information that can help to achieve better quality of care (13).

To relate patients’ experiences to an operationalized and comparable framework, in 2000 the World Health Organization (WHO) introduced the concept of responsiveness (7). Responsiveness has been defined as a measure of how well the health system responds to the population's non-medical expectations when interacting with the system (8). Although many studies have evaluated health care responsiveness as part of the WHO Multi-Country Service Study (MCSS) (7), to our knowledge the application of the concept of responsiveness specifically to the mental health care system has been limited (13). Given the context specific organization of health systems and the relevance of cultural norms in mental health, it is important to have a valid instrument that is easy for mental health service providers in Iran to both understand and use. The aim of our study was to adapt the original form of the Health System Responsiveness Questionnaire, developed by the WHO, to the mental health care system in Iran, by determining the validity and reliability of this new version.

## Methods

### Scale development

To evaluate the general health care system responsiveness on a national level, WHO developed and validated a questionnaire by using a comprehensive review of existing instruments and field tests of new and adapted items. The questionnaire measures responsiveness for general inpatient and outpatient care in eight domains (7, 14). The English version Health System Responsiveness Questionnaire was translated into Farsi by the first author. The translated version was adapted based on the findings of our previous qualitative studies in which we evaluated the applicability of the health system responsiveness concept to the Iranian mental health system (15, 16). As a result, a new domain of effective care was added, the domain of prompt attention was divided into two new labeled domains – access to care and attention – moreover, the domains choice of health care providers and autonomy were integrated, and some new questions were added to existing domains. Table 1 compares the domains covered in both the original and the adapted questionnaires. A bilingual expert back-translated the Farsi modified version to English. An expert panel modified the back-translated questionnaire until all members confirmed that it was comparable to the original English version. The approved version was back-translated to Farsi and two bilingual experts independently confirmed the translation. Items regarding demographic information were also added to the final version of the questionnaire.

**Table 1:** Domains covered in WHO and Farsi responsiveness questionnaire

The domains covered in WHO responsiveness questionnaire	The domains covered in Farsi responsiveness questionnaire
Confidentiality (To handle patients’ information confidentially)	Confidentiality (To handle patients’ information confidentially)
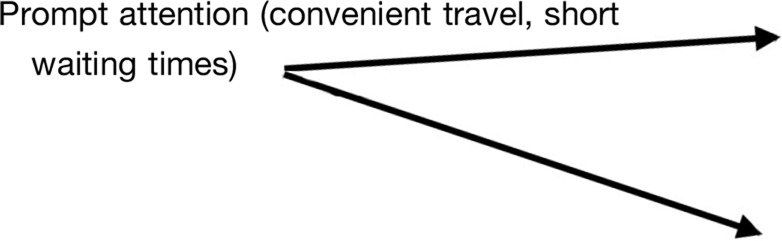	Access to care (Acceptable care provided as soon as needed by patient)
	Attention (Close and affable dialogue between mental health workers and patients, to attend to with deep understanding to the patients, having enough time to ask questions, proactive and careful follow-up of the process of treatment, mental health care providers show they understand how patients feel about their problem)
Dignity (respectful treatment, communication)	Dignity (Showing respect when treating patients, non-stigmatizing treatment, taking patients problems and complaints seriously, maintaining individuality and to recognize patients’ individual needs and characteristics)
Clear communication (Listening, enough time for questions, clear explanations)	Clear communication (To provide patients with understandable information about their problem, to provide information about patient problems in a comprehensible manner)
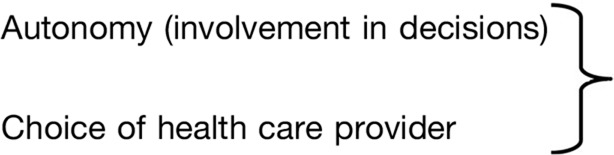	Autonomy (Services and providers can be chosen freely, to be able to participate in therapeutic decisions and processes, Patient/provider relationship at the same level)
–	Effective care (To provide practical and continuous care advice in congruence with patient norms and values, to provide services commensurate with costs such as time and money by same familiar person)
Quality of basic amenities (surroundings)	Quality of basic amenities (To be treated in clean, informal and friendly places)

### Setting and design

In Tehran, Iran's capital, mental health services are organized in terms of catchment areas. Each of the four public medical universities is responsible in terms of providing and supervising mental health services for a defined catchment area with specific geographical boundaries. The corresponding public medical university also supervises the existing private mental hospitals and outpatient clinics.

The approved Farsi version of the Mental Health System Responsiveness Questionnaire (MHSRQ) was tested in a pilot study carried out in two outpatient centers with 20 participants. Based on these findings, the wording of several items was revised for clarification. The final questionnaire consisted of 40 questions representing eight domains. The domain ‘access to social support’ was excluded from the questionnaire because inpatient cases were not included in this study.

Between January and April 2013, a cross-sectional survey was implemented in all nine outpatient public mental health clinics, distributed in different city regions (north, south, east, west, and central); private psychiatric clinics were not included. A non-random sample of 500 mentally ill patients attending the selected clinics was recruited. The number of participants was calculated using the number of items entered into the factor analytic procedure. As a general rule, 10 subjects are necessary for each variable in factor analysis (17). The number of participants assigned to each clinic was proportional to the total volume of patients attending the clinics during the previous 3 months. All participants were diagnosed as mentally ill based on a professional psychiatric evaluation. The inclusion criteria for participating in the study were 1) being an adult (18–65 years old), 2) receiving outpatient care during past 12 months, and 3) according to their clinical record, being in remission phase of their disorder and mentally capable to follow the interview. The type of participants’ mental disorder was not considered as inclusion criteria because health care experiences relate more to the health services functioning than to the patient's current diagnosis (13, 18).

### Data collection procedure

Through participation in a 4-hour training session, 10 interviewers with a bachelor degree in psychology learned about the background and objectives of the study. In addition, the respondent selection procedures and interview process were explained to participants. On the basis of the pilot study, it was decided that the interviewers would read the questions to those participants with 5 years or less of formal education.

Patients attending the public mental health clinics were recruited after being approached by interviewers and asked for their consent to participate. All participants were interviewed in mental health clinics and each interview lasted approximately 45–50 min. Before the interview, each participant was informed about the objectives of the study, explaining that the completion of the questionnaire was voluntary and their identification would be protected, as the data files were anonymous. The Ethical Committee and Research Council of the University of Social Welfare and Rehabilitation Sciences, Tehran approved the study protocol.

### Statistical analysis

The questionnaire consists of 40 items related to eight domains. The items dealing with distance or time duration were calculated based on the actual hours or days it took from them. Then we categorized items 6,100 and 6,104 into four responses as 1) (less than 1 day), 2) (1–7 days), 3) (8–30 days), and 4 (more than 1 month). Items 6,101, 6,105 and 6,106 were calculated with the same principle and categorized as 1 (less than 30 min), 2 (30–60 min), 3 (1–3 hours), and 4 (more than 3 hours). The items related to quality of basic amenities (6,170, 6,171, 6,172) ranged from 1 (very good), 2 (good), 3 (moderate), 4 (bad), and 5 (very bad). The rest of the items in the questionnaires ranged from 1 (Always), 2 (Often), 3 (Sometimes), and 4 (Never). For rating questions (overall) the response categories 5 (very bad), 4 (bad), 3 (moderate), 2 (good), and 1 (very good) were used.

Failure to include all participants’ data in the analysis may bias the results. Our first approach was to investigate the missing data and assess whether respondents had substantial difficulties in answering the questions. This was done calculating the item-missing rate, as the percentage of non-response to an item and the average across sections of the questionnaire. A missing rate of 5% or less was considered ignorable, whereas items with more than 20% (19) missing were considered problematic.

Reliability of the questionnaire was checked with internal consistency assessment methods. Consistency of the entire scale was assessed using the Cronbach's alpha coefficient. For each item, the alpha is given if the item is deleted. Other internal consistency assessment methods included the item-test correlation (the correlation of the item score with the average of items within a domain) and the item-rest correlation coefficients (the correlation of the item score with domain average that excludes the item from the equation). Other studies suggested that the item-test correlations exceed 0.5 and Cronbach's alpha exceed 0.7 (20).

To evaluate the construct validity, we focus on the internal structure of the questionnaire, particularly on the dimensionality and homogeneity of items (questions) hypothesized to represent one domain. As the WHO (7) had already established the factor structure of the instrument, confirmatory factor analysis (CFA) was used to assess the construct validity of the new instrument. CFA followed Jöreskog's guidelines for the analysis of ordinal data (21). Diagonal weighted least-squares estimation was applied to polychoric correlations that were based on the asymptotic covariance matrix. Although, according to the WHO, there is no strict cut-off to describe the power of the association of the variance, the closer to −1 or +1, the stronger the unidimensionality of the construct (7). However, Hair et al., revealed that for a practical significance, loading factors ±0.3 are of minimal significance, loadings ±0.4 are considered important, and ±0.5 indicate significant loading (22). The models were evaluated by means of Bentler–Satorra chi-square score, root mean square error of approximation (RMSEA) (23), goodness-of-fit index (GFI), and adjusted goodness-of-fit index (AGFI), where the values of RMSEA less than 0.05 indicate a close fit, values in the range of 0.05 to 0.08 indicate fair fit, and that values above 0.1 indicate poor fit. For GFI, AGFI, and comparative fit index [CFI], values exceeding 0.90 indicated a good fit of the model to the data. CFI and incremental fit index were also reported (24, 25), where values equal to or greater than 0.90 denote an acceptable fit to the model (26, 27). Confirmatory factor analyses were performed using LISREL 8.8.

## Results

The results of the descriptive statistics of demographic characteristics of participants showed that of the 500 patients enrolled in the study, 38% were female and 62% were male. The majority of participants were in the 25–35 year old age group (33.4%). About 24% had 5 years or less of formal education and 28.7% were unemployed. All participants revealed that they had more than one time experience of using the services during past 6 months, and 96% more than two times. The majority of participants (52.7%) revealed that they belong to the middle social class and 92.8% of participants had access to medical insurance.

### Response rate and missing

The item-missing rate is reported in Table 2. All items met the pre-established criteria for feasibility of less than 20% missing. The access domain and its related questions showed the highest average item-missing rate (5.2%).

**Table 2:** Average item missing rates for mental health responsiveness questionnaire

Domain	Items	Item missing rate (%)
Attention	How often did the mental health care professionals listen to what you said with full attention	0.2
	How often your statements were deeply understood by the mental health care professionals	0.2
	How often did the mental health care professionals show courtesy and affection towards you	0.4
	How often did the mental health professionals spend enough time in asking you questions	0.2
	How often the mental health professionals were accurately and actively involved in following up your treatment process	0.8
	How often did you feel that the mental health professionals have understood you	0.2
	How often were you provided enough time by the mental health professionals	0.4
	Overall score	0.6
		0.4*
Dignity	How often did mental health professionals treat you with respect	0.2
	How often did the office working in the mental care services treat you with respect	0.4
	How much attention did the mental health professionals paid specifically into your needs and characteristics	0.4
	Overall score	0.4
		0.4*
Clear communication	How often did mental health professionals explain things in a way you could understand	0.2
	How often … explain things and issues related to your mental health in detail for you	0.4
	Overall score	0.4
		0.3*
Autonomy	How big a problem if any, was it to get an appointment with the mental health professional of your choice	0.2
	How big a problem, if any, was it to get to use other health services other than the one you usually went to	0.4
	How often did the mental health professionals made you actively involved the decision making process	0.6
	How often did the mental health professionals ask your permission before the start of treatment process or laboratory tests	0.8
	How often did the health made you feel that you have the competence, capability and the power to participate in the decision making process	0.2
	Overall score	0.6
		0.5*
Effective care	How often were the instructions and treatment recommendations that you received conforms to your norms and values	1.2
	How often did you constantly seek consultation on the same familiar mental health professional	1.2
	How often the center or mental health professionals were properly coordinated with each other	4.0
	How were the services you received worthy for the money and the time spent	0.2
	Overall score	0.6
		0.8*
Access to care	How long did you have to wait to get mental health care services	6.8
	How long did you stay in the waiting room	6.4
	How often did you get care as soon as you wanted	4.2
	How much distance would you have to undertake in order to get mental health care	9.3
	Overall score	0.2
		5.2*
Confidentiality	How often were the interviews remained confidential	1
	Mental health professionals keep your personal information confidential	1.2
	Overall score	1
		1*
Quality of basic	How would you assess the whole quality of the waiting room	0.4
amenities	How would you assess the cleanliness of this place	0.4
	How would you assess its warmth and friendly environment	0.4
	Overall score	0.4
		0.4*

* Average item-missing rate.

### Reliability

The findings presented in Table 3 show the item-test correlation, the item-rest correlation, and the Cronbach's alpha coefficient (consistency of the entire scales). Overall, the results of the item correlation test were in the acceptable range with a Cronbach's alpha coefficient >0.70 for six of the eight domains. Access to care (*α*=0.56), and effective care (*α*=0.66) were the worst performing domains.

**Table 3:** Item correlation and alpha coefficients for domain questions on level of responsiveness

Item	Short item description	Item-test correlation	Item-rest correlation	Alpha if item deleted
Access				
s6100	How long did you have to wait to get mental health care services	0.51	0.24	0.53
s6101	How long did you stay in the waiting room	0.58	0.34	0.50
s6102	How often did you get care as soon as you wanted	0.46	0.26	0.52
s6104	How long does it take to complete laboratory tests, x-ray examination and EEG	0.55	0.28	0.52
s6105	How long did you have to wait for laboratory tests, x-ray examination and EEG	0.57	0.40	0.48
s6106	How much distance would you have to undertake in order to get mental health care	0.50	0.21	0.55
s6107	Rate access	0.50	0.29	0.51
Test scale				0.56
Communication				
s6110	How often did mental health professionals explain things in a way you could understand	0.94	0.86	0.86
s6111	How often … explain things and issues related to your mental health in detail for you	0.94	0.86	0.86
s6112	Rate communication	0.90	0.78	0.92
Test scale				0.92
Confidentiality				
s6120	How often were the interviews remained confidential	0.90	0.78	0.75
s6121	How often mental health professionals keep your personal information confidential	0.90	0.79	0.75
s6122	Rate confidentiality	0.85	0.64	0.89
Test scale				0.86
Dignity				
s6130	How often did mental health professionals treat you with respect	0.75	0.58	0.64
s6131	How often did the office working in the mental care services treat you with respect	0.72	0.50	0.67
s6132	How much attention did the mental health professionals paid specifically into your needs and characteristics	0.75	0.46	0.71
6133	Rate dignity	0.77	0.56	0.64
Test scale				0.73
Attention				
s6140	How often did the mental health care professionals listen to what you said with full attention	0.78	0.72	0.89
s6141	How often your statements were deeply understood by the mental health care professionals	0.81	0.75	0.89
s6142	How often did the mental health care professionals show courtesy and affection towards you	0.70	0.61	0.90
s6143	How often did the mental health professionals spend enough time in asking you questions	0.79	0.71	0.89
s6144	How often the mental health professionals were accurately and actively involved in following up your treatment process	0.74	0.66	0.90
s6145	How often did you feel that the mental health professionals have understood you	0.85	0.80	0.89
s6146	How often were you provided enough time by the mental health professionals	0.78	0.69	0.90
s6147	Rate attention	0.77	0.69	0.90
Test scale				0.91
Autonomy				
s6150	How big a problem if any, was it to get an appointment with the mental health professional of your choice	0.40	0.22	0.77
s6151	How big a problem, if any, was it to get to use other health services other than the one you usually went to	0.64	0.30	0.83
s6152	How often did the mental health professionals made you actively involved the decision making process	0.79	0.68	0.66
s6153	How often did the mental health professionals ask your permission before the start of treatment process or laboratory tests	0.82	0.72	0.65
s6154	How often did the health made you feel that you have the competence, capability and the power to participate in the decision making process	0.79	0.68	0.67
s6155	Rate autonomy	0.75	0.65	0.69
Test scale				0.75
Effective care				
s6160	How often were the instructions and treatment recommendations that you received conforms to your norms and values	0.58	0.27	0.69
s6161	How often did you constantly seek consultation on the same familiar mental health professional	0.69	0.41	0.61
s6162	How often the center or mental health professionals were properly coordinated with each other	0.56	0.33	0.64
s6163	How often were the services you received worthy for the money and the time spent	0.67	0.48	0.58
s6164	Rate effectivity of care	0.81	0.67	0.50
Test scale				0.66
Quality of basic amenities				
s6170	How would you assess the whole quality of the waiting room	0.89	0.80	0.86
s6171	How would you assess the cleanliness of this place	0.90	0.80	0.86
s6172	How would you assess its warmth and friendly environment	0.82	0.68	0.91
s6173	Rate quality of basic amenities	0.91	0.84	0.85
Test scale				0.90

### Construct validity

**Fig. 1:**
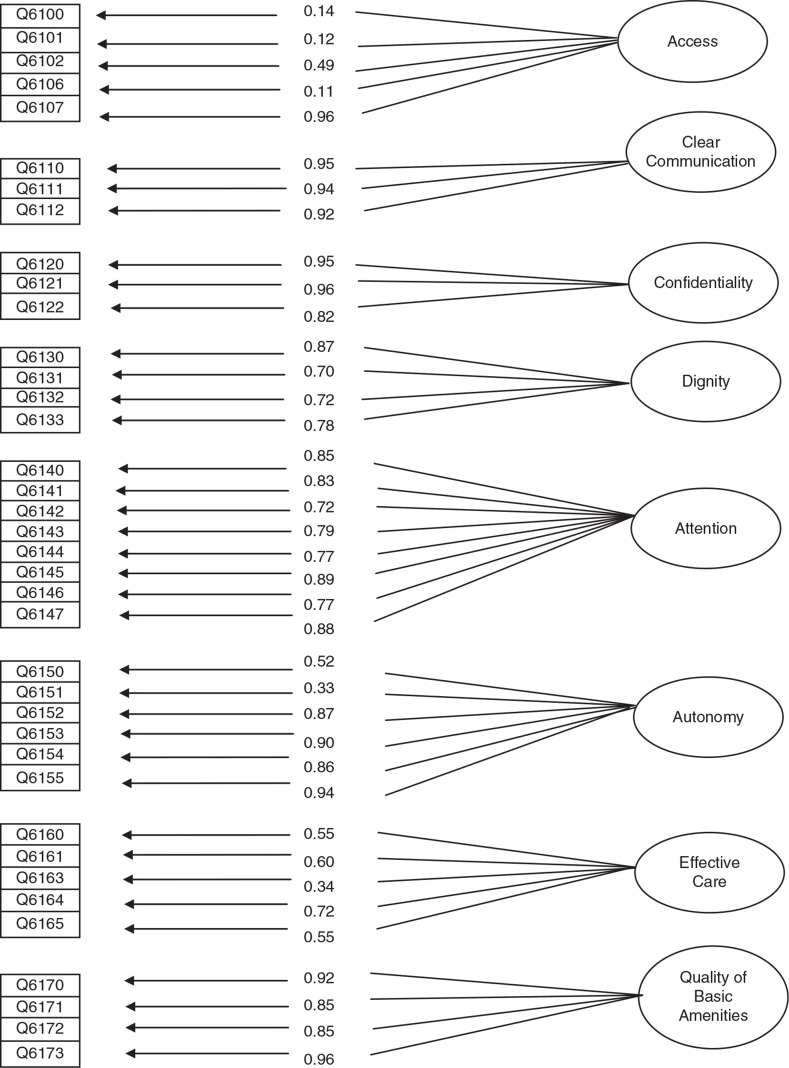
Factor model obtained for the questionnaire based on confirmatory factor analysis.

Figure 1 presents the results of the factor analysis based on the responses of the participants in the survey. The numbers indicate the factor loadings on the latent variable that represent the amount of variance that an item has in common with that latent variable. Three items related to the domain of access did not perform well: 1) the item on ‘the length of time from requesting care to receiving it’, 2) the item about ‘length of time staying in waiting room before receiving the needed mental health service’, and 3) and the item regarding the ‘distance to reach the mental health care service’. Chi-square was calculated as 79.3 (df=566) which was not significant. Modification attempts were conducted in a step-wise procedure; at each step the items that had higher residuals than 0.8 (Theta-delta) were identified and that with the highest residual was eliminated. Two items, namely 6,151 (residual=0.894,) and 6,162 (residual=0.88) were removed based on this criteria. Correlations among eight factors are given in Table 4. Fit indices for this model were all indicative of an acceptable model (CFI=0.99, GFI=0.97, IFI=0.99, AGFI=0.97).

**Table 4:** Correlation among eight domains of mental health system responsiveness questionnaire

Domain	Comm.	Access	Conf.	Dignity	Attention	Autonomy	Effect.	Quality.
Comm.	1							
Access	0.34	1						
Conf.	0.35	0.34	1					
Dignity	0.63	0.42	0.57	1				
Attention	0.73	0.28	0.41	0.65	1			
Autonomy	0.43	0.46	0.32	0.49	0.50	1		
Effect	0.47	0.52	0.32	0.51	0.43	0.42	1	
Quality	0.31	0.39	0.14	0.35	0.36	0.34	0.41	1

## Discussion

The findings of this study show acceptable reliability and validity properties for the Farsi version of the MHSRQ. Fit indices of the overall model were good (GFI, AGFI, CFI>0.9), although the domain of access did not perform well in the psychometric evaluation.

With the exception of the access domain (5.2%), the missing rates reported were lower than 1% for all domains. However, even the missing rate for the access domain was lower than the pre-established cut-off level of 20% (19). The worst performing items of this domain were those concerning the time it takes to reach a mental care clinic. Problems with these items have also been noted in the short form of the MCSS questionnaire for general health patients (7). One reason for problems with this domain is that the questions in this domain might have still been difficult for respondents to understand. Mental health users usually attend care services several times during 1 year. Therefore, they might have found it difficult to remember the waiting time at the different visits. Technical modifications and wording revision of these items might be useful to overcome this problem.

The internal consistency of the questionnaire was good. The figures are similar to the classical psychometric assessment for the original responsiveness instrument (7), reinforcing its reliability. Our findings show that the responsiveness domains with the highest Cronbach's alpha were communication, attention, and quality of basic amenities. The latter showed a high alpha coefficient in the original version of the responsiveness instrument as well (7). The high Cronbach's alpha might indicate that the questions related to the domain were referring to similar issues and measuring the same aspects of the domain. Access (*α*=0.56) was the worst performing domain. Inconsistency of items related to this domain might be due to the different formulation of the questions. Most items related to this domain are composed of sets of measures that are not intrinsically correlated, such as waiting time and distance (28). To improve the internal consistency of these items one suggestion is to separate these questions and create new domains. The other domain showing low Cronbach's alpha (0.66) was effective care. However the alpha coefficient of this domain is not very far from the acceptable level (0.7). It should be considered that effective care is a newly formed domain and the wording of items related to this domain may not be clear enough for respondents.

The validity of the questionnaire was tested by focusing on the internal structure, in particular the dimensionality of the questions representing a domain. Although the results generally confirmed the structure of the responsiveness domains, three items related to the access domain showed loading factors less than the acceptable level of 0.3 (22). These items dealt again with time and distance in which numerical responses were more appropriate for reporting them. Because our analytical approach was suitable for analyzing ordinal responses (21), the continuous responses were transformed into categorical variables. Accordingly, this might explain the low correlation between these categorical responses and the actual continuous time/distance variables originally reported by respondents.

This study also includes certain limitations. The psychiatric diagnosis of participants was ignored arguing that patients’ experience with the mental health system is not related to their current diagnosis. Although, we are aware that there are some literature suggesting that patient satisfaction with the system could be affected by their diagnosis (29, 30). Following WHO instructions, first time patients were included. However, few times of experiencing mental health services could make it difficult for patients to give an accurate answer to some questions. Because there was not a previously validated instrument in Iran, it was not possible to make a direct comparison. Inpatient mental health users were not included because of the difficulties in accessing this group while they are in the remission phase of their illness and thus cognitively capable to participate in the study. Therefore, the domain related to access to a social support network, which is only relevant to inpatient care, was not included in the study.

## Conclusions

This study has reported the feasibility, reliability, and validity of the WHO instrument used to assess mental health system responsiveness in Iran. A low item missing rate indicates that it is feasible to apply the instrument in Iran. The reliability and internal consistency of the questionnaire was acceptable in general, although some items showed lower item correlation than others. With exception to the access domain, a validity investigation also showed good results for all domains of the questionnaire and consistent responses in general. Further steps will include additional research to overcome some of the limitations of the present study. The future application of the Farsi version of the MHSRQ will positively contribute to mental health system improvements in Iran.
